# Predator-induced transgenerational plasticity in animals: a meta-analysis

**DOI:** 10.1007/s00442-022-05274-w

**Published:** 2022-11-01

**Authors:** Kirsty J. MacLeod, Chloé Monestier, Maud C. O. Ferrari, Katie E. McGhee, Michael J. Sheriff, Alison M. Bell

**Affiliations:** 1 Department of Biology, Lund University, Sölvegatan 37, 223 62 Lund, Sweden; 2 School of Natural Sciences, Bangor University, Deiniol Road, Bangor, LL57 2UR UK; 3 Department of Evolution, Ecology and Behavior, Carle R. Woese Institute for Genomic Biolog, University of Illinois, 505 S. Goodwin Ave., Urbana, IL 61801 USA; 4 Department of Biomedical Sciences, WCVM, University of Saskatchewan, 52 Campus Drive, S7N 5B4, Saskatoon, SK Canada; 5 Department of Biology, The University of the South, Sewanee, TN 37375 USA; 6 Biology Department, University of Massachusetts Dartmouth, Dartmouth, MA 02747 USA

**Keywords:** Parental effect, Maternal effect, Developmental plasticity, Intergenerational inheritance, Predation

## Abstract

There is growing evidence that the environment experienced by one generation can influence phenotypes in the next generation via transgenerational plasticity (TGP). One of the best-studied examples of TGP in animals is predator-induced transgenerational plasticity, whereby exposing parents to predation risk triggers changes in offspring phenotypes. Yet, there is a lack of general consensus synthesizing the predator–prey literature with existing theory pertaining to ecology and evolution of TGP. Here, we apply a meta-analysis to the sizable literature on predator-induced TGP (441 effect sizes from 29 species and 49 studies) to explore five hypotheses about the magnitude, form and direction of predator-induced TGP. Hypothesis #1: the strength of predator-induced TGP should vary with the number of predator cues. Hypothesis #2: the strength of predator-induced TGP should vary with reproductive mode. Hypothesis #3: the strength and direction of predator-induced TGP should vary among offspring phenotypic traits because some traits are more plastic than others. Hypothesis #4: the strength of predator-induced TGP should wane over ontogeny. Hypothesis #5: predator-induced TGP should generate adaptive phenotypes that should be more evident when offspring are themselves exposed to risk. We found strong evidence for predator-induced TGP overall, but no evidence that parental predator exposure causes offspring traits to change in a particular direction. Additionally, we found little evidence in support of any of the specific hypotheses. We infer that the failure to find consistent evidence reflects the heterogeneous nature of the phenomena, and the highly diverse experimental designs used to study it. Together, these findings set an agenda for future work in this area.

**Supplementary Information:**

The online version contains supplementary material available at 10.1007/s00442-022-05274-w.

## Introduction

Transgenerational plasticity (TGP, aka environmental parental effects) occurs when the environment experienced in one generation influences traits in the subsequent generation(s). TGP is the across-generation version of within-generational plasticity and has attracted considerable attention from both theoretical (Räsänen and Kruuk 2007; Badyaev and Uller 2009; Jablonka and Raz 2009; Bonduriansky and Day 2009; Kuijper and Hoyle 2015; Wolf and Wade 2016) and empirical perspectives (e.g., reviewed in Uller 2012; Bell and Hellmann 2019; Moore et al. 2019). A wide range of environmental conditions—climate change, temperature, pollutants, diet, etc.—has been shown to trigger TGP, with one of the best-studied types of TGP occurring in response to predation risk (e.g. Agrawal et al. 1999; Shine and Downes 1999; Sheriff et al. 2010, 2020; Storm and Lima 2010; Peacor et al. 2012; Walsh et al. 2015; Bell et al. 2016; Donelan and Trussell 2018; Monteforte et al. 2020). Predator-induced TGP is particularly well-studied because of its potential to scale up to ecologically relevant population level effects (Peacor et al. 2012, 2020; Sheriff et al. 2020); non-consumptive effects of predators on the next generation are likely to have important consequences for prey population demography and individual life-history variation (Clinchy et al. 2013; Sheriff et al. 2020; Peacor et al. 2020). Moreover, the rich existing literature on predator–prey ecology provides a solid framework for generating adaptive hypotheses about why, when, and how predator-induced TGP is expected to evolve and to take particular forms (Table 1).

**Table 1:** Hypotheses and moderators within the two themes

Theme	Hypothesis #	Moderator	Categories
Moderators in parents	Hypothesis #1: Predator-induced TGP is stronger when parents experience multiple predator cues	Predator cue type	Single visual cue (e.g. models, predator separated by an impermeable barrier blocking other cue types)
			Single auditory cue (e.g. predator calls in playback)
			Single chemical cue (e.g. kairomones, alarm substance)
			Multicomponent (any combination of > 1 of the above)
			Predator presence (live, visible predator in same medium as prey)
	Hypothesis #2: Predator-induced TGP is stronger in viviparous species than oviparous species	Reproductive mode	Oviparous, viviparous
Moderators in offspring	Hypothesis #3: Labile traits are less susceptible to predator-induced TGP than stable traits	Offspring trait category	Size/mass
			Growth/development (e.g. early life growth, hatching or development time)
			Physiology (e.g. cortisol/corticosterone levels, body condition, mitral cell activity)
			Behaviour (e.g. learning, response to predator cues, activity level, feeding rate)
	Hypothesis #4: Predator-induced TGP weakens as offspring age (i.e., strongest effects when measured at birth)	Age category at offspring measurement	Independent embryo
			Birth (within 3 days of hatching/parturition)
			Juvenile/maturity
	Hypothesis #5: Predator-induced TGP is apparent when offspring experience high-risk conditions	Risk of offspring testing environment	High-risk predator environment (e.g. predator’s cue presence, response to predator directly measured)
			Low-risk environment (e.g. control environment, conspecific presence, novel object)

Such hypotheses can be broadly grouped based on whether they pertain mostly to sources of variation at the parental level or outcomes at the offspring level. For example, numerous studies on predator–prey interactions have shown that animals mount strong antipredator responses when they detect multiple cues indicative of predation risk (Bouwma and Hazlett 2001; Ward and Mehner 2010; Reynolds and Bruno 2013). To the extent that multiple predator cues reflect greater certainty about risk and elicit a larger parental response, we tested Hypothesis #1: predator-induced TGP should be stronger when parents are exposed to multiple predator cues from the same predator, or an actual live predator (which presumably provides multiple cues), rather than just a single type of cue (e.g. auditory, visual, chemical).

While the strength of the risk signal should influence predator-induced TGP, so should the extent to which mothers and offspring are connected prior to birth. Viviparous mothers are in intimate contact with their embryos up to the time of birth and thus might continuously pass on cues to offspring over a longer time period (Love et al. 2013). In contrast, oviparous mothers primarily pass on physiological cues to offspring during the egg formation period only and these signals might wane over time (Vassallo et al. 2014; Carter et al. 2018). This is relevant in the context of predator-induced TGP because predation risk can presumably trigger a glucocorticoid stress response in mothers (Wingfield et al. 1998), which could influence not only her physiology and behaviour but also potentially the developmental environment of her offspring, especially if the offspring is exposed to elevated glucocorticoids while in utero (Seckl 2004; Meaney et al. 2007). Indeed, glucocorticoid levels in eggs have been shown to decrease over time in oviparous species and thus such a signal may become diminished as *in-ovo* embryos develop (Paitz et al. 2011). A recent meta-analysis in amniotic vertebrates demonstrated stronger, and more detrimental effects, of maternally-mediated glucocorticoids during development on offspring traits in viviparous relative to oviparous species (MacLeod et al. 2021). Therefore, we set up Hypothesis #2: Predator-induced TGP is expected to be stronger in viviparous compared to oviparous species.

In addition to sources of variation at the parental level, variation at the offspring level can contribute to variation in predator-induced TGP outcomes. Factors such as the traits measured in offspring, offspring age, and the environment offspring experience, can shape predator-induced TGP outcomes and, as such, generates three additional hypotheses. Hypothesis #3: the strength and direction of predator-induced TGP should vary among offspring phenotypic traits because some traits are more plastic than others. The possibility that some offspring traits are more susceptible to predator-induced TGP than others is relevant in the context of predation risk responses because animals respond to predators with morphological, physiological, and behavioural defences (Peckarsky et al. 2008; Sheriff and Thaler 2014). For example, some authors have suggested that relatively slow-changing traits, such as morphological traits, are more likely to be influenced by transgenerational plasticity compared to more labile traits, such as behavioural or physiological traits, which are often influenced by current environmental conditions (Tariel et al. 2020). Moreover, a large number of offspring traits have been examined in the TGP literature, and they broadly fall into the categories of morphology, physiology, and behaviour. Therefore, this breadth provides an excellent opportunity to test this hypothesis.

Hypothesis #4: parental effects are expected to weaken as offspring age, for several reasons. For example, parental effects may wane over development as offspring become better at acquiring their own information about current environmental conditions and making phenotypic adjustments based on their own experience (Snell-Rood et al. 2015; Stamps and Krishnan 2017). Additionally, the parental environment during reproduction is more likely to predict the offspring’s early-life environment, and to become less predictable as time goes on, especially for long-lived animals or those in unstable environments (Burgess and Marshall 2014). In the context of predator-induced TGP, predator *and* prey behaviour contributes to this waning relevance of prenatal signals: predation risk varies as predators move around the landscape, *and* offspring dispersal distances can greatly vary, thus, there may be substantial spatial–temporal differences in predation risk experienced by the offspring as compared to their parents. Weakening parental effects over development have been found in other recent meta-analyses (Moore et al. 2019; Yin et al. 2019; MacLeod et al. 2021) and in the quantitative genetics literature generally (Wilson and Réale 2006).

Hypothesis #5: predator-induced TGP and parental effects may not become apparent unless offspring are exposed to predation risk themselves. The adaptive matching hypothesis posits that parental effects adaptively prepare offspring for their future environment (Mousseau and Fox 1998; Uller 2008; Sheriff and Love 2013). According to this hypothesis, offspring traits induced by parental predator exposure are likely most relevant in high-risk environments and, thus, may not be expressed in benign control environments (Uller et al. 2013; Sheriff et al. 2018). This may be particularly true for labile trait categories such as behaviour and physiology, where an increase in refuge use or stress hormone responsiveness may not be apparent unless offspring are exposed to predation risk themselves.

Here, we apply a meta-analytical framework to the fast-growing literature on predator-induced TGP (441 individual effect sizes, from 29 species, and 49 studies) to test the limits of our understanding of TGP in general and in the context of predator–prey ecology in particular. After assessing whether parental experience of predation risk during reproduction results in consistent effects on offspring traits across studies and taxonomic groups, we use this framework to test these five hypotheses. We broadly group these hypotheses according to whether they concern sources of variation (moderators) in parents (Hypothesis #1: cues of predation risk to which the parents were exposed and Hypothesis #2: whether they are oviparous or viviparous) or whether they concern sources of variation (moderators) in offspring (Hypothesis #3: offspring trait types, Hypothesis #4: offspring stage of development, and Hypothesis #5: offspring risk environment). We note that there are many other moderators in both the parental and offspring generations that would be of great interest to investigate (for example related to timing of exposure during development), however, we had insufficient data to test such moderators meaningfully across such a broad range of taxa. Our meta-analysis thus focuses on those moderators for which we had sufficient data and that generated broad testable hypotheses as outlined above. This is the first attempt, to our knowledge, to quantitatively examine predator-induced TGPs across species synthesizing both predation risk and TGP theory.

## Methods

### Literature search

We searched for studies concerning predator-induced TGP published up to August 2020 on ISI Web of Science and Google Scholar using the following search string: maternal effect* OR paternal effect* OR parental effect* OR transgenerational OR carry over OR anticipatory OR embryonic learning OR embryo* expos* AND: predat*. All titles were scanned for relevance (i.e. must relate to studies of parental predator exposure). Resulting from this, we found 636 unique published studies (see Fig. S1 for full PRISMA diagram). Abstracts were scanned for the following inclusion criteria: (1) parents and offspring were not domesticated species or laboratory strains; (2) offspring phenotype was measured (if a study included both grandparental and parental effects, we only extracted parent–offspring effects); (3) parents were exposed to a predation risk cue and any effects were generated by such predator exposure or cues, and not by direct manipulation of “stress” (e.g. treatment with glucocorticoids); and (4), subjects were exposed to predator cues in the context of breeding (i.e. not including studies of the effects of early- or mid-life predator exposure on subsequent future reproduction) and with a controlled experiment (i.e. control and treatment groups). Note that we focused on studies of sexually mature individuals that were exposed to predation cues near reproduction, and this included studies in which exposure occurred pre- and during ovulation, pre-fertilization, as well as post-fertilization. We did not include studies in which parents were exposed as immature individuals, or studies in which offspring might also have been exposed to cues while parents were providing parental care. Although this eliminated studies of early parental exposure and adjustments to parental care, this allowed us to examine the importance of mode of reproduction (*Hypothesis #2*) more directly and it linked the parental experience more closely to early offspring experience (*Hypotheses # 3*).

We rejected 465 papers at the abstract screening level; the remaining 171 papers were read in full, after which a further 122 papers were rejected as they did not reach our criteria (full details in Supplementary Material S1). Our final dataset comprised 49 studies.

### Data preparation

We extracted means and standard errors of offspring traits in response to parental predator treatments from eligible studies and according to moderators of interest. If values were not presented in the text or supplementary data, means and errors were extracted from figures using the R package *metaDigitise* (Pick et al. 2018), WebPlotDigitizer software (Rohatgi 2020), or the program GraphClick (version 3.0.3, 2012, Arizona Software), or by contacting authors directly. Moderators (predator cue type, reproductive mode, offspring trait measured and age at measurement) are described in full in Table 1. Papers were assigned randomly to authors for data extraction but to maximize objectivity, individual authors did not extract data from papers which they had authored; instead, data from papers that were written by one of the authors were extracted by another member of the team. A full list of offspring traits on which data were gathered, and trait categories these were placed in (i.e. morphology; growth and development; physiology; behaviour), is presented in Supplementary Material S2.

### Statistical analysis and data summary

Our full dataset on predator-induced TGP comprised 441 individual effect sizes, from 29 species, and 49 studies. Some species were disproportionately represented (e.g. of the 57 effect sizes from birds, 40 were from *Parus major*). A phylogenetic tree of the species represented was constructed using supertrees from the Open Tree of Life (Rees and Cranston 2017) in the *rotl* and *ape* packages (Michonneau et al. 2016; Paradis and Schliep 2019) in R (R Core Team 2018) (see Supplementary Material S3 for tree and breakdown of species including N individual effect sizes, and % of the total N effect sizes). Effect sizes (Standardized Mean Differences, SMD, also known as Hedge’s *g*) were calculated using the package *metafor* (Viechtbauer 2010). Control group sample sizes were corrected for studies that compared more than one treatment group to a shared control group to account for repeated information. The direction of the parental effect was estimated by the extent to which trait values were relatively larger or smaller as a function of parental exposure. For some traits, e.g. latency to emerge, the scale was inverted to match biological interpretation (i.e. larger values indicate a greater phenotypic response). All models were constructed using the *R* package *metafor* (Viechtbauer 2010).

Egger’s test (using variance *vi* as moderator) showed no significant funnel asymmetry of the data on Predator-induced TGP (*F*_1,439_ = 0.20, *p* = 0.65), i.e. there was no evidence of over-reporting of large effect sizes either through publication bias or effects of small studies. Heterogeneity among data was high (tested in an overall model without moderators, *I*^2^ [total]: 96.2%). This was mostly due to high within-study heterogeneity (*I*^2^ [trait]: 64.9%), while there was relatively lower heterogeneity among studies (*I*^2^ [study]: 31.23%) and among species (*I*^2^ [species]: < 0.1%).

Before testing the five specific hypotheses, we first assessed if there was overall evidence that predator exposure in one generation shifted the average trait value in the next generation (i.e., that there was general evidence of predator-induced TGP). Although this is almost taken for granted in the literature (Tariel et al. 2020), it has not been rigorously quantified or established. It has been suggested that increased trait variation is expected under stress (Hoffmann and Hercus 2000) and/or when the future environment is dangerous or uncertain (diversified bet hedging) (Crean and Marshall 2009). Therefore, we also examined whether parental predator exposure resulted in higher variance in offspring traits (Tariel et al. 2020). To examine these questions, we first ran a basic model without moderators to determine the overall effect of parental predator exposure on all offspring traits (*N* = 441 effect sizes). We accounted for study ID, species, as well as individual trait as random terms (i.e. each effect size is assigned an ID, giving an individual trait-level random term to account for within-study effect size variance additional to sampling error). Phylogeny was controlled for by including a relatedness matrix derived from the phylogenetic tree (using Grafen’s method to compute branch lengths) as a random effect in the model. To assess evidence for the overall strength of predator-induced TGP, we additionally estimated the absolute value of Hedges' *g* (|g|) by repeating the basic model without moderators as described above, using a Bayesian meta‐analytic meta‐regression model in R in the *MCMCglmm* package (Hadfield 2010), and applying posterior distributions of parameters from Gaussian models to the folded normal distribution to obtain mean estimates and credible intervals for absolute magnitudes (i.e. ‘analyse and transform’ sensu Morrissey 2016, analysis code adapted from Noble et al. 2017). MCMC chains were run for 510,000 iterations with a 10,000 iteration burn in and a thinning interval of 1000. In total across the three chains, we ran 1,500,000 iterations sampling 1500 iterations from the posterior distribution. We also tested whether parental predator exposure resulted in greater phenotypic variation in offspring relative to the control group by performing a meta-analysis of variance. We calculated the variance effect size statistic lnCVR (the natural logarithm of the coefficients of variation between the control and treatment groups) and its measurement error variance using the equation detailed in Nakagawa et al.(2015). Note that this statistic could not be calculated for non-ratio data (i.e. where means were < 0, *N* = 6 effect sizes). The meta-analytical model (*N* = 435) was as described before, again including study ID, species, and individual trait-level random terms.

To test hypotheses about moderators influencing predator-induced TGP, we ran a global model which simultaneously tested the effect of the following variables corresponding to Hypotheses #1–4 (see Table 1 for full moderator level descriptions): predator cue type, reproductive mode, offspring trait category, and age at measurement. As before, we included the phylogeny described before and study ID, species, and individual trait-level random terms.

Hypothesis #5 posits that predator-induced TGP differs depending on offspring risk environment (i.e. exposure to the same parents’ predator exposure or to a new one will reveal predator-induced TGP in offspring, while a low-risk environment that does not include any predation risk will not). To investigate this, we ran a separate model using a subset of the data containing only studies that specifically tested traits in high or low predation risk environments (including studies where offspring were raised, as well as tested, in high/low risk environments), or responses to predators (i.e. high risk) (*N* = 149 effect sizes). This model contained only offspring risk environment as a variable (i.e. absolute SMD ~ offspring risk environment). Random effects, including phylogeny, were specified as in previous models. We additionally conducted sensitivity analyses to test the influence of non-independence of data points from the same study by calculating moderator coefficients and confidence intervals derived from robust variance estimation from models (Hedges et al. 2010). Where robust variance estimation did not substantially alter results (see Results), these models are reported only in Supplementary material S4.

## Results

### Overall patterns

**Fig. 1:**
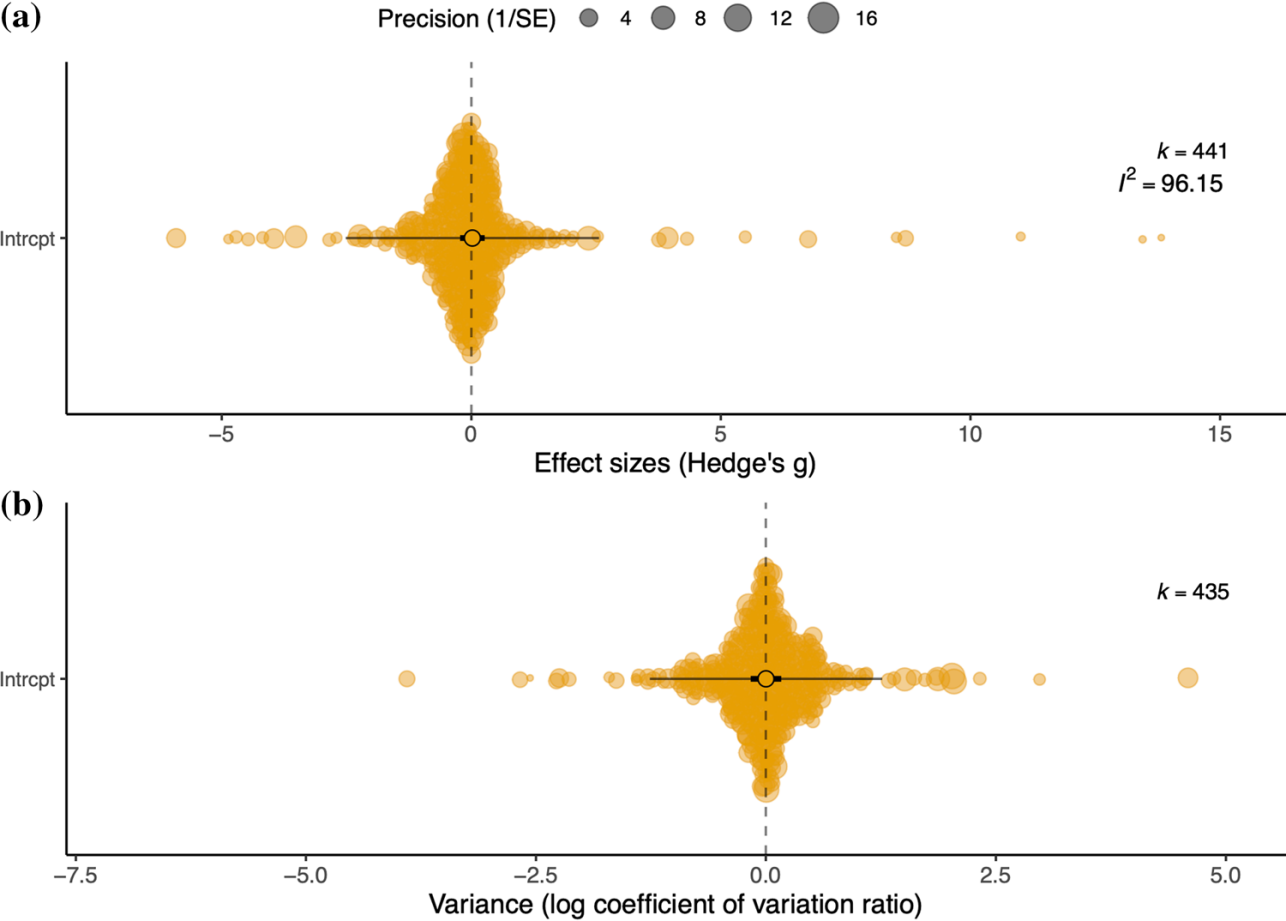
Orchard plots showing the overall **a** direction of Predator-induced TGP effects (Hedge’s *g*), as well as **b** the effect of Predator-induced TGP on trait variance (lnCVR, values > 0 represent datapoints where treatment group variance exceeded control group variance). Model estimates ± SE are depicted as overlaid black open circles ± thick bar, with a thin bar representing the prediction intervals. Dashed line indicates zero (i.e. no effect) and k values indicate the number of effect sizes

There was a wide range of effect sizes across the dataset (from − 5.91 to 13.82). Disregarding direction, the mean effect size (± s.e.) was 0.73 ± 0.07, in the region of a medium-to-large effect size (Cohen 1977). This indicates there is strong evidence for predator-induced TGP overall: although there was no evidence that parental predator exposure causes offspring traits to change in a particular direction (est 0.02, CI 95% -0.23, 0.27; Table 2, Fig. 1a), the estimated value for absolute effect sizes (|g|) was large (est 1.06, CI 95% 0.96, 1.40). There was no evidence that parental predator exposure consistently increases variance in offspring traits, i.e. diversified bet-hedging (est 0.002, CI 95% − 0.16, 0.17; Table 2, Fig. 1b).

**Table 2:** Results of random effects meta‐analyses models testing the effect of parental exposure to predation risk on all offspring traits (Hedges g; *N* = 441 effect sizes)

Effect size	*k*	Meta-analytic mean ± s.e	95% CI	*P*	*I*^2^ Total (%)	*I*^2^ Study (%)	*I*^2^ Species (%)	*I*^*2*^ Obser. (%)
Hedges *g*	441	0.02 ± 0.13	− 0.23, 0.27	0.87	96.15	31.23	< 0.01	64.93
lnCVR	233	0.002 ± 0.08	− 0.16, 0.17	0.98	94.14	3.12	35.74	55.27

Model heterogeneity (% *I*^2^) also shown

### Hypothesis #1: predator-induced TGP should be stronger when parents experience multiple predator cues

Whether parents experienced a single predator cue, multiple predation cues, or a live predator did not change the strength of predator-induced TGP (Table 3). However, sensitivity analysis via robust variance estimation showed that when accounting for within-study covariance of effect sizes, cue type had moderately more influence on predator-induced TGP, with auditory cues having positive effects on offspring traits and chemical cues having moderately negative effects (Supplementary Table S3a).

**Table 3:** Full model testing the effects of moderators on predator-induced TGP in offspring traits (Hedges g; *N* = 441 effect sizes)

	Est ± s.e	95% CI (ci.lb, ci.ub)	*T*	*P*	Anova test of moderator
Intercept	1.13 ± 0.98	− 0.79, 3.05	1.16	0.25	
**Offspring trait**					*F*_3,429_ = 5.26, *P* = 0.001**
Behaviour/performance					
Growth/development	0.54 ± 0.28	− 0.02, 1.09	1.91	0.06	
Physiology	− 0.16 ± 0.27	− 0.70, 0.37	− 0.60	0.55	
Size/mass	− 0.46 ± 0.18	− 0.82, − 0.11	− 2.54	0.01	
**Age at measurement**					*F*_3,429_ = 0.74, *P* = 0.53
Embryo					
Birth	0.06 ± 0.49	− 0.90, 1.02	0.12	0.90	
Juvenile	− 0.22 ± 0.46	− 1.12, 0.68	− 0.48	0.63	
Maturity	0.02 ± 0.46	− 0.88, 0.93	0.05	0.95	
**Reproductive mode**					*F*_1,429_ = 2.25, *P* = 0.13
Oviparous					
Viviparous	0.48 ± 0.32	− 0.15, 1.12	1.50	0.13	
**Cue type**					*F*_4,429_ = 1.50, *P* = 0.20
Auditory					
Chemical	− 1.36 ± 0.87	− 3.07, 0.35	− 1.56	0.12	
Visual	− 0.76 ± 0.89	− 2.51, 1.00	− 0.84	0.40	
Multicomponent	− 0.71 ± 0.87	− 2.43, 1.01	− 0.81	0.42	
Predator presence	− 0.79 ± 0.87	− 2.51, 0.92	− 0.91	0.36	

Significance tests of all moderators individually are presented in the table, and an overall test of all moderators combined, and model heterogeneity (% *I*^2^), are also shown

Test of moderators: *F*_11,429_ = 2.12, *P* = 0.02; *I*^2^ total = 96.27% (*I*^2^_paper_ = 26.77%, *I*^2^_species_ = 8.22%, *I*^2^_effectsize_ = 61.27%)

### Hypothesis #2: predator-induced TGP should be stronger in viviparous species than oviparous species

There was no indication that oviparous and viviparous prey species differed in predator-induced TGP effects (Table 3).

### Hypothesis #3: offspring traits should vary in susceptibility to predator-induced TGP

**Fig. 2:**
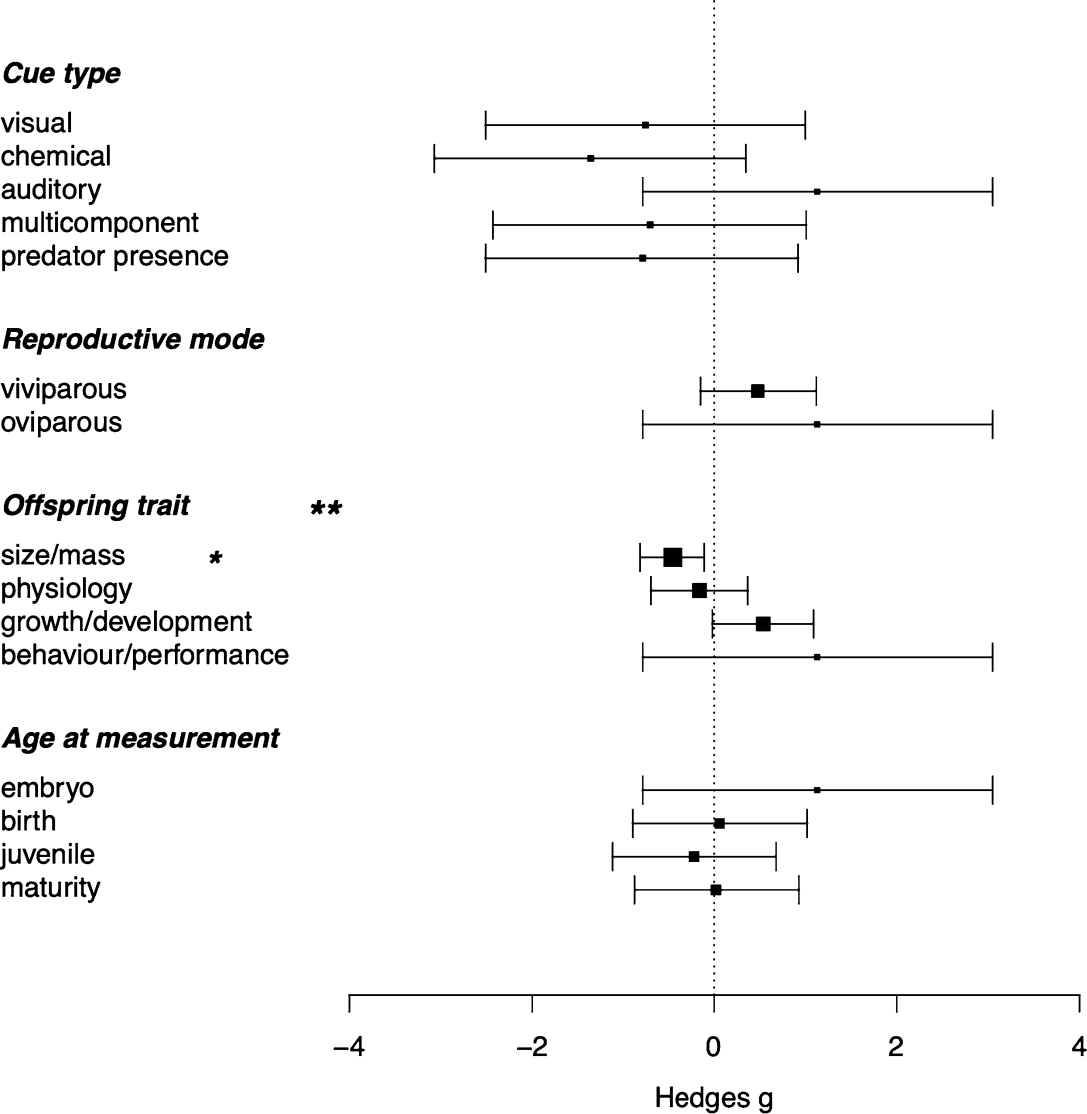
Forest plot showing the influence of the type of offspring trait measured on Predator-induced TGP (*N* = 441 effect sizes). Model estimates ± 95% CIs are depicted as points and bars with arrows. The size of the estimate point relates to the precision of the point (1/SE)

Predator-induced TGP outcomes differed among offspring traits (Fig. 2; Table 3): effects on offspring growth and developmental traits were positive while effects on size and mass traits were significantly negative (overall effect of trait category *F*_3,429_ = 5.26, *P* = 0.001, Table 3; Fig. 2). There was no evidence of strong effects on either physiological or behavioural traits (Table 3; Fig. 2). Sensitivity analysis using robust variance estimation indicated that, to an extent, these effects were influenced by covariance of effect sizes within studies. Offspring trait was less important accounting for clustering of data according to paper ID (*F*_3,37_ = 1.45, *P* = 0.24; full model results in Supplementary Material S4). However, estimates show that negative effects of parental predator exposure on mass remain strong even accounting for clustering of data (95% CI − 0.92, − 0.01).

Offspring survival was included with growth and development traits but the number of studies measuring survival was very low (*N* = 5). This low number cautions against making general inferences about the effects of predator-induced TGP on survival specifically, however, of these five studies, two demonstrate that parental predator-exposure increases offspring survival while three show a negative effect on offspring survival.

### Hypothesis #4: predator-induced TGP should weaken as offspring age (i.e. strongest effects should occur when measured at birth)

There was no evidence that predator-induced TGP weaken as offspring age (Table 3).

### Hypothesis #5: predator-induced TGP should be more apparent when offspring experience high-risk conditions

**Fig. 3:**
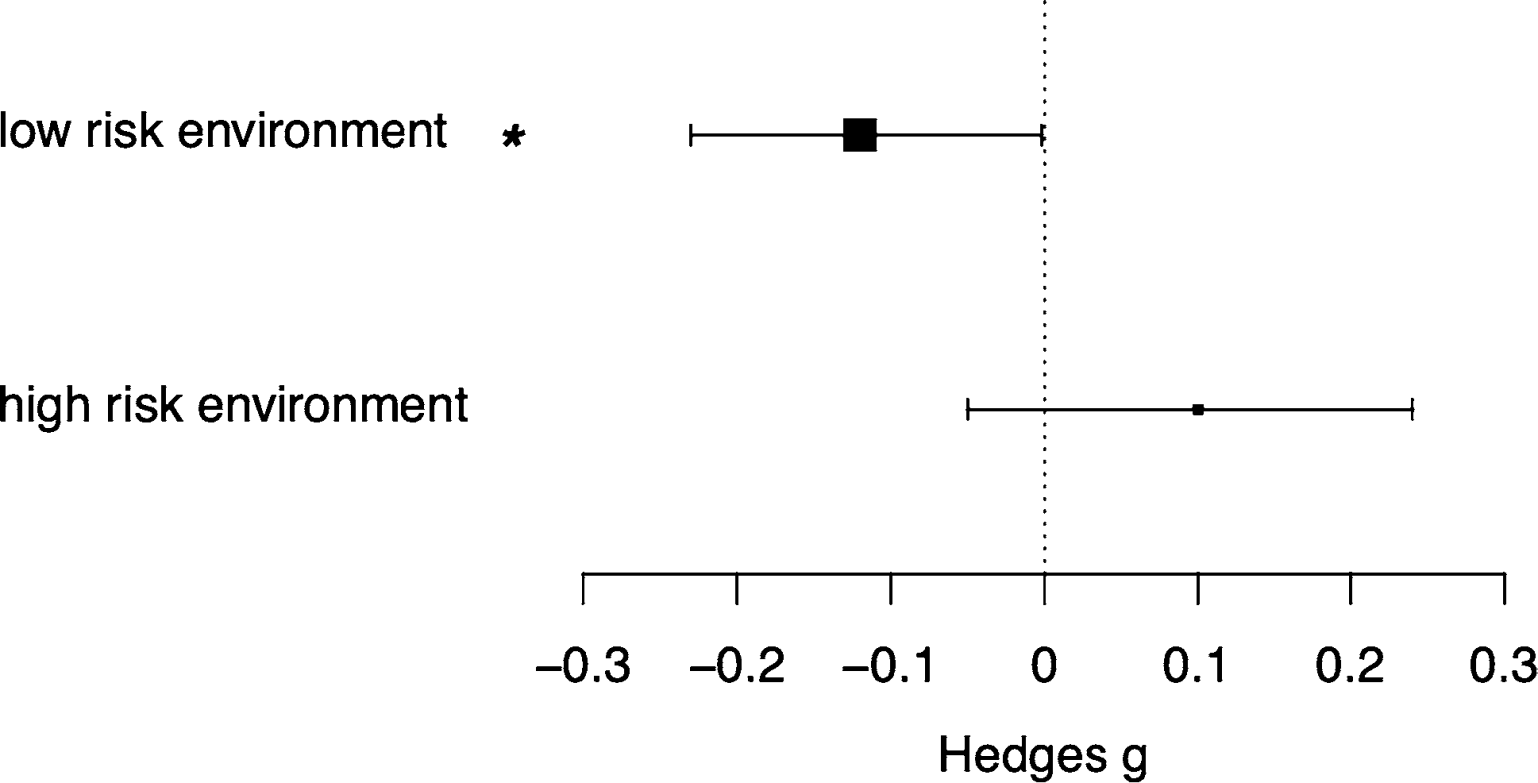
Forest plot showing influence of offspring risk environment on Predator-induced TGP (*N* = 149 effect sizes, from 13 studies). Model estimates ± 95% CIs are depicted as points and bars with arrows. The size of the estimate point relates to the precision of the point (1/SE)

In the subset of studies that specifically tested offspring traits in high or low risk environments or in the response of offspring to predators (*N* = 13 studies and 149 effect sizes), offspring traits measured in low-risk environments were more negatively affected than those measured in high-risk environments (low-risk est − 0.12, 95% CI − 0.23, − 0.002; high-risk est 0.10, 95% CI − 0.05, 0.25; Fig. 3). However, this difference was not significant (test of moderator *F*_1,147_ = 1.78, *P* = 0.18, Fig. 3).

## Discussion

Here, we exploited what has become a sizable literature documenting predator-induced TGP (49 papers and 441 effect sizes) to provide the first quantitative synthesis of this growing field. We sought to determine whether we can make generalizations about the ways in which predation risk experienced in one generation can influence traits in the next generation. Overall, evidence for predator-induced TGP was strong, with no evidence of publication bias: on average, across a wide range of taxa and experimental designs, offspring of predator-exposed parents consistently differed from offspring of unexposed parents. This provides an important confirmation that the widespread nature of predator-induced TGP effects as has been posited regularly in the literature (Tariel et al. 2020) is indeed likely. However, there was little evidence in support of any of the generalizable hypotheses that we tested.

The main consistent pattern that was detected across the wide range of studies included in this meta-analysis is that predator-induced TGP varied among offspring trait categories in two specific ways. First, predator-exposed parents tended to produce smaller, lighter offspring. The negative effect of parental predator exposure on offspring body size could be mediated by prenatal exposure to stress-related hormones during pregnancy or in-ovo, which has been shown to decrease offspring birth weight in humans and other vertebrate animal models (Cottrell and Seckl 2009; Eberle et al. 2021), though broader effects of prenatal hormone exposure on offspring size and mass in wild vertebrates (glucocorticoids: MacLeod et al 2021) and invertebrates (Miyashita and Adamo 2020) remain less clear. From an ultimate perspective, producing smaller offspring in response to predation risk may be an adaptive response by mothers to increase their own reproductive value when adult survival is low (Moore et al. 2016) by reducing their own investment under stress (Sheriff et al. 2009; Berghänel et al. 2017). Second, we also found that predator-exposed parents tended to produce offspring that developed and grew faster. Accelerated development and postnatal growth may be a general adaptive response to increased predation risk because eggs and small juveniles are often the most vulnerable to predation (Biro et al. 2005; Urban 2007). Increased growth rate and reaching adult size faster may confer advantages such as escaping gape-limited predators, or accelerating metamorphosis and escape of risky habitats (Dahl and Peckarsky 2003; Biro et al. 2005; Urban 2007). Note, however, that whether production of large vs small offspring is viewed as adaptive from the parental and/or offspring perspective will depend on the species and the context; for example, producing offspring larger at birth can also be advantageous in avoiding gape-limited predators (Sharda et al. 2021).

Overall, we found no consistent evidence for the five hypotheses that we tested. Importantly, the number of studies and effect sizes included in this meta-analysis was relatively large, and comparable in statistical power to similar recent meta-analyses (e.g. MacLeod et al. 2021; Dougherty et al. 2022). Below, we discuss three possible related reasons for this lack of support: (1) The current dataset is too heterogeneous and confounded; (2) The metric used to evaluate predation risk is not consistent; i.e., predator–prey relationships are not consistent; (3) The phenomenon is complex (e.g. environmental predictability is almost always unknown) and species-specific (e.g. substantial life history variation).

### 1. The current dataset is too heterogeneous and confounded

Similar to other meta-analyses on transgenerational plasticity and parental effects (Uller et al. 2013; Moore et al. 2019; Eyck et al. 2019; Yin et al. 2019; MacLeod et al. 2021), this meta-analysis detected considerable heterogeneity in the dataset. That is, there was substantial variation in the magnitude and direction of predator-induced TGP: the effects of parental predator exposure on offspring traits ranged from negative (decreased trait values) to positive (increased trait values), with several studies also reporting no strong effects in either direction. Confounds in the dataset are likely to have contributed to the dataset’s heterogeneity, thereby influencing the ability to detect consistent effects of moderating variables. For example, reproductive mode was strongly confounded with taxonomic differences (e.g. nearly all mammals are placentatrophic and viviparous while all birds are oviparous), which could make it difficult to detect an overall effect of reproductive mode. Future studies comparing species differing in reproductive mode within a taxonomic group, e.g. reptiles varying along a continuum of ovo- and viviparity (MacLeod et al. 2021), could be insightful.

Moreover, studies in this meta-analysis employed a wide variety of experimental designs and study systems. The importance of this is highlighted by our exploration of cue type. Some studies applied predator cues to parents for a relatively short period of time (Giesing 2010; McGhee et al. 2012; Roche et al. 2012; Elliott et al. 2016), while other studies applied predator cues to parents for the entire reproductive period (Dzialowski et al. 2003; Bian et al. 2005; Mikulski and Pijanowska 2010; Sentis et al. 2017), for example. If the duration of exposure is confounded with the type of cue (visual versus olfactory, for example), and taxa (terrestrial versus aquatic, for example), and if duration, cue type and taxa all matter, then these patterns are likely to be obscured when comparing across studies. Focused studies deliberately manipulating both the duration of exposure and type of cue in carefully-selected taxa could help to clarify the relative importance of specific factors.

### 2. The predation risk evaluation has no common metric

An outstanding challenge for studies in this area is that predation risk effects, and thus predator-induced TGP, are highly system-specific and depend upon not only the particular predator and prey species but the context under which such interactions occur. For example, the same prey individual may perceive (and respond) to predation risk from an ambush predator very differently than a cursorial predator, and any such response is likely to depend on many aspects within the environment, such as food availability and refuges (Schmitz et al. 1997; Lima and Steury 2005; Kelleher et al. 2021). Predator cues may also be intrinsically or contextually more or less predictive of actual predation risk (i.e. certain cue types are more or less reliable, or reliability depends on prey species or other environmental factors), adding to the differences in parental perception of or response to risk. For example, we focused on the influence of one cue versus several based on the idea that redundancy should increase reliability and therefore response to cues (Bouwma and Hazlett 2001; Ward and Mehner 2010; Reynolds and Bruno 2013); however, it is of course possible that cue types vary in information value to prey thus also generating variation in reliability and response, but this is likely to be highly system and species-specific (indeed, no single cue type produced stronger effects in our dataset, possibly due to this heterogeneity). Thus, determining the relative magnitude of perceived risk in the parental generation and comparing across a common metric is challenging.

Unsurprisingly, our dataset revealed considerable breadth in methodological approaches to tackling this problem, with “parental predator exposure” treatments varying in cue type and number (which we attempted to account for), as well as other temporal and spatial factors that likely combine to determine parental and offspring perception of predation risk (which we were unable to account for, e.g. proximity, the duration of exposure, social context, habitat variation, etc.). Furthermore, the response in the offspring’s phenotype may be greatly altered by such contextual factors, e.g., offspring may exhibit increased growth if parents are exposed to gape-limited predators, but may have reduced growth and activity if refuges are present. We are not the first to note this methodological variation and the problems it poses for determining broad patterns (Schmitz 2005; Moll et al. 2017). We suggest future research attempt to provide a common metric for parental perception of risk (e.g., glucocorticoid hormones, metabolic rate, food intake, etc.) that will facilitate comparisons across diverse systems.

### 3. The phenomena is complex and species-specific

Another distinct possibility is that predator-induced TGP (and other forms of TGP) is too complex and context-dependent for us to make generalizations across such broad groups. For example, it is already known that parental exposure to risk affects offspring traits in both a sex-dependent (e.g. sex of parent exposed and sex of offspring (Mashoodh et al. 2009; St-Cyr et al. 2017; Hellmann et al. 2020a, b; McGhee et al. 2021) and size-dependent (McGhee et al. 2012, 2021) manner. Detecting consistent results when comparing even the same trait across species appear challenging; the functional significance of the same trait, e.g. latency to move (freezing), could depend on context. Freezing may be an important anti-predator behaviour for some prey species exposed to predators that detect prey via motion (McGhee et al. 2013); however, this same behaviour may be detrimental for other predator–prey interactions, where reducing latency to move or increasing flight-initiation-distance may instead be the best anti-predator response (Samia et al. 2013). Making things more complicated still is recognition that success in a predator encounter is rarely tied to a single trait and thus measuring predator-induced TGP on isolated offspring traits at particular moments in time might not provide a comprehensive picture of the adaptive nature of predator-induced TGP. If what really matters is how traits change together, then examining the effects of parents’ experience with predation risk on entire suites of traits in offspring is a promising way forward.

It is also likely that the outcome of predator-induced TGP is strongly species-specific, which limits the ability to detect general patterns. We highlight two examples. First, species that are highly vulnerable to predation risk may have evolved to be highly responsive to any indication of risk (i.e., the smoke detector principle, Nesse 2001), rather than titrating their response according to the magnitude or quality of risk cues (Sheriff et al. 2018). Therefore species’ differences in their evolutionary histories with predation risk might have contributed to the failure to detect support for the hypothesis that predator-induced TGP is strongest in response to multiple cues (Hypothesis #1). Second, species differences in how habitat use changes over ontogeny might have resulted in a lack of support for the hypothesis that predator-induced TGP wanes over development (Hypothesis #4). In species with distinct ontogenetic habitat shifts (e.g. aquatic to terrestrial), parents' experience during reproduction may only be relevant to the environment their offspring are likely to experience later in life. Adaptive predator-induced TGP might be expected to grow stronger rather than weaker with age in such animals. On the other hand, in species subject to predatory threats which are constant across the species' ecology and ontogeny (i.e. the environment is highly stable over time and space), plasticity in general may be reduced as sensitivity to environmental cues becomes less vital (Reed et al. 2010). Such variation in environmental stability may exist even at the population, rather than species, level. Focused comparisons of predator-induced TGP across closely related species (e.g. with different developmental modes or differing evolutionary histories with predation risk), or across populations in different specific ecologies are warranted.

Finally, while statistically non-significant, we show some evidence that the riskiness of the offspring’s environment could influence whether the outcomes of predator-induced TGP are “positive” or “negative” for offspring traits, i.e., that the offspring environment can influence whether such trait changes are adaptive or not. Yet, a key finding of our paper is that this sort of context-dependency was infrequently tested, likely limiting our ability to detect truly consistent patterns. Similar to previous studies that have tested the adaptive matching hypothesis using meta-analysis (e.g. Uller et al. 2013), we found that estimation of the expected degree of autocorrelation between the parental and offspring environment, an important determinant of the likely adaptive benefit of TGP (Kuijper and Hoyle 2015), was most often lacking.

## Conclusions

Conceptually, the study of predator-induced TGP benefits from strong theory in both predator–prey ecology and TGP. Predator-induced TGP has clear ecological relevance, and the topic has attracted considerable attention from both theoretical and empirical perspectives. One of the key “take home messages” of this meta-analysis is that predator-induced TGP is widespread and common: despite the highly heterogenous nature of the dataset, our results confirm that predator-induced TGP occurs across this wide range of taxa, experimental designs, and predation threats.

This meta-analysis also highlights important gaps in our knowledge and considerations for future work. Indeed, the other key “take home message” of this meta-analysis is that our understanding of predator-induced TGP is clearly incomplete. We examined five hypotheses based on the extensive literature in this area, and marshalled an impressive dataset to test them, but did not find support for any of them possibly because of the heterogeneity of studies and complexity of the phenomena. Another possibility is that many of the hypotheses evaluated in this meta-analysis are motivated by adaptive reasoning, and it is possible that some, if not most, predator-induced TGP is nonadaptive, which leads to highly idiosyncratic results. Given that there are multiple mechanisms by which parental experiences influence the next generation (e.g. microbiome, habitat selection, hormones, epigenetic changes, etc.) and the mechanism will influence how and which traits are affected, further consideration of the mechanism of transmission is likely to be highly insightful.

## Supplementary Information

Below is the link to the electronic supplementary material.

Supplementary file1 (DOCX 334 KB)

## Data Availability

Data are available at 10.5281/zenodo.7241419.

## Code availability

 Code is available at 10.5281/zenodo.7241419.

## References

[CR1] Agrawal AA, Laforsch C, Tollrian R (1999). Transgenerational induction of defences in animals and plants. Nature.

[CR3] Badyaev AV, Uller T (2009). Parental effects in ecology and evolution: mechanisms, processes and implications. Philos Trans R Soc Lond B Biol Sci.

[CR5] Bell AM, Hellmann JK (2019). An integrative framework for understanding the mechanisms and multigenerational consequences of transgenerational plasticity. Annu Rev Ecol Evol Syst.

[CR6] Bell AM, McGhee KE, Stein LR (2016). Effects of mothers’ and fathers’ experience with predation risk on the behavioral development of their offspring in three spined sticklebacks. Curr Opin Behav Sci.

[CR7] Berghänel A, Heistermann M, Schülke O, Ostner J (2017). Prenatal stress accelerates offspring growth to compensate for reduced maternal investment across mammals. Proc Natl Acad Sci.

[CR8] Bian J, Wu Y, Liu J (2005). Effect of predator-induced maternal stress during gestation on growth in root voles Microtus oeconomus. Acta Theriol (Warsz).

[CR9] Biro PA, Post JR, Abrahams MV (2005). Ontogeny of energy allocation reveals selective pressure promoting risk-taking behaviour in young fish cohorts. Proc R Soc B Biol Sci.

[CR10] Bonduriansky R, Day T (2009). Nongenetic inheritance and its evolutionary implications. Annu Rev Ecol Evol Syst.

[CR96] Bouwma P, Hazlett BA (2001). Integration of multiple predator cues by the crayfish Orconectes propinquus. Anim Behav.

[CR11] Burgess SC, Marshall DJ (2014). Adaptive parental effects: the importance of estimating environmental predictability and offspring fitness appropriately. Oikos.

[CR13] Carter AW, Bowden RM, Paitz RT (2018). Evidence of embryonic regulation of maternally derived yolk corticosterone. J Exp Biol.

[CR14] Clinchy M, Sheriff MJ, Zanette LY (2013). Predator-induced stress and the ecology of fear. Funct Ecol.

[CR15] Cohen J (1977). Statistical power analysis for the behavioral sciences.

[CR16] Crean AJ, Marshall DJ (2009). Coping with environmental uncertainty: dynamic bet hedging as a maternal effect. Philos Trans R Soc B Biol Sci.

[CR17] Cottrell EC, Seckl JR (2009). Prenatal stress, glucocorticoids and the programming of adult disease. Front Behav Neurosci.

[CR18] Dahl J, Peckarsky BL (2003). Developmental responses to predation risk in morphologically defended mayflies. Oecologia.

[CR19] Donelan SC, Trussell GC (2018). Synergistic effects of parental and embryonic exposure to predation risk on prey offspring size at emergence. Ecology.

[CR20] Dougherty LR, Skirrow MJA, Jennions MD, Simmons LW (2022). Male alternative reproductive tactics and sperm competition: a meta-analysis. Biol Rev.

[CR21] Dzialowski AR, Lennon JT, O’Brien WJ, Smith VH (2003). Predator-induced phenotypic plasticity in the exotic cladoceran Daphnia lumholtzi. Freshw Biol.

[CR22] Eberle C, Fasig T, Brüseke F, Stichling S (2021). Impact of maternal prenatal stress by glucocorticoids on metabolic and cardiovascular outcomes in their offspring: a systematic scoping review. PLoS ONE.

[CR23] Elliott KH, Betini GS, Dworkin I, Norris DR (2016). Experimental evidence for within- and cross-seasonal effects of fear on survival and reproduction. J Anim Ecol.

[CR24] Eyck HJF, Buchanan KL, Crino OL, Jessop TS (2019). Effects of developmental stress on animal phenotype and performance: a quantitative review. Biol Rev.

[CR25] Giesing ER (2010). Mothers transfer information via eggs: effect of mothers’ experience with predators on offspring.

[CR27] Hadfield JD (2010). MCMC methods for multi-response generalized linear mixed models: The **MCMCglmm**
*R* Package. J Stat Softw.

[CR28] Hedges LV, Tipton E, Johnson MC (2010). Robust variance estimation in meta-regression with dependent effect size estimates. Res Synth Methods.

[CR29] Hellmann JK, Bukhari SA, Deno J, Bell AM (2020). Sex-specific plasticity across generations I: Maternal and paternal effects on sons and daughters. J Anim Ecol.

[CR30] Hellmann JK, Carlson ER, Bell AM (2020). Sex-specific plasticity across generations II: grandpaternal effects are lineage specific and sex specific. J Anim Ecol.

[CR31] Hoffmann AA, Hercus MJ (2000). Environmental stress as an evolutionary force. Bioscience.

[CR32] Jablonka E, Raz G (2009). Transgenerational epigenetic inheritance: prevalence, mechanisms, and implications for the study of heredity and evolution. Q Rev Biol.

[CR33] Kelleher V, Hunnick L, Sheriff MJ (2021). Risk-induced foraging behavior in a free-living small mammal depends on the interactive effects of habitat, refuge availability, and predator type. Front Ecol Evol.

[CR34] Kuijper B, Hoyle RB (2015). When to rely on maternal effects and when on phenotypic plasticity?. Evol Int J Org Evol.

[CR35] Lima SL, Steury TD, Barbosa P, Castellanos I (2005). Perception of predation risk: the foundation of non-lethal predator-prey interactions. Ecology of predator-prey interactions.

[CR36] Love OP, McGowan PO, Sheriff MJ (2013). Maternal adversity and ecological stressors in natural populations: the role of stress axis programming in individuals, with implications for populations and communities. Funct Ecol.

[CR37] MacLeod KJ, While GM, Uller T (2021). Viviparous mothers impose stronger glucocorticoid-mediated maternal stress effects on their offspring than oviparous mothers. Ecol Evol.

[CR38] Mashoodh R, Sinal CJ, Perrot-Sinal TS (2009). Predation threat exerts specific effects on rat maternal behaviour and anxiety-related behaviour of male and female offspring. Physiol Behav.

[CR39] McGhee KE, Barbosa AJ, Bissell K (2021). Maternal stress during pregnancy affects activity, exploration and potential dispersal of daughters in an invasive fish. Anim Behav.

[CR40] McGhee KE, Pintor LM, Bell AM (2013). Reciprocal behavioral plasticity and behavioral types during predator-prey interactions. Am Nat.

[CR41] McGhee KE, Pintor LM, Suhr EL, Bell AM (2012). Maternal exposure to predation risk decreases offspring antipredator behaviour and survival in threespined stickleback. Funct Ecol.

[CR42] Meaney MJ, Szyf M, Seckl JR (2007). Epigenetic mechanisms of perinatal programming of hypothalamic-pituitary-adrenal function and health. Trends Mol Med.

[CR43] Michonneau F, Brown JW, Winter DJ (2016). rotl: an R package to interact with the Open Tree of Life data. Methods Ecol Evol.

[CR44] Mikulski A, Pijanowska J (2010). When and how can Daphnia prepare their offspring for the threat of predation?. Hydrobiologia.

[CR45] Miyashita A, Adamo SA (2020). Stayin’alive: endocrinological stress responses in insects. Advances in invertebrate (neuro) endocrinology.

[CR46] Moll RJ, Redilla KM, Mudumba T (2017). The many faces of fear: a synthesis of the methodological variation in characterizing predation risk. J Anim Ecol.

[CR47] Morrissey MB (2016). Meta-analysis of magnitudes, differences and variation in evolutionary parameters. J Evol Biol.

[CR48] Monteforte S, Cattelan S, Morosinotto C (2020). Maternal predator-exposure affects offspring size at birth but not telomere length in a live-bearing fish. Ecol Evol.

[CR49] Moore MP, Riesch R, Martin RA (2016). The predictability and magnitude of life-history divergence to ecological agents of selection: a meta-analysis in live bearing fishes. Ecol Lett.

[CR50] Moore MP, Whiteman HH, Martin RA (2019). A mother’s legacy: the strength of maternal effects in animal populations. Ecol Lett.

[CR51] Mousseau TA, Fox CW (1998). The adaptive significance of maternal effects. Trends Ecol Evol.

[CR53] Nakagawa S, Poulin R, Mengersen K (2015). Meta-analysis of variation: ecological and evolutionary applications and beyond. Methods Ecol Evol.

[CR54] Nesse RM (2001). The smoke detector principle. Natural selection and the regulation of defensive responses. Ann N Y Acad Sci.

[CR55] Noble DWA, Stenhouse V, Schwanz LE (2017). Early thermal environments and developmental plasticity in reptiles: a systematic review and meta-analysis. Biol Rev.

[CR56] Paitz RT, Bowden RM, Casto JM (2011). Embryonic modulation of maternal steroids in European starlings (*Sturnus vulgaris*). Proc R Soc B Biol Sci.

[CR57] Paradis E, Schliep K (2019). ape 5.0: an environment for modern phylogenetics and evolutionary analyses in R. Bioinformatics.

[CR58] Peacor SD, Barton BT, Kimbro DL (2020). A framework and standardized terminology to facilitate the study of predation-risk effects. Ecology.

[CR59] Peacor SD, Pangle KL, Schiesari L, Werner EE (2012). Scaling-up anti-predator phenotypic responses of prey: impacts over multiple generations in a complex aquatic community. Proc Biol Sci.

[CR60] Peckarsky BL, Abrams PA, Bolnick DI (2008). Revisiting the classics: considering nonconsumptive effects in textbook examples of predator-prey interactions. Ecology.

[CR61] Pick JL, Nakagawa S, Noble DWA (2018) Reproducible, flexible and high throughput data extraction from primary literature: The metaDigitise R package. BioRxiv

[CR62] R Core Team (2018) R: A language and environment for statistical computing. R Foundation for Statistical Computing, Vienna, Austria

[CR63] Räsänen K, Kruuk LEB (2007). Maternal effects and evolution at ecological time-scales. Funct Ecol.

[CR64] Reed TE, Waples RS, Schindler DE, Hard JJ, Kinnison MT (2010). Phenotypic plasticity and population viability: the importance of environmental predictability. Proc Roy Soc b: Biol Sci.

[CR65] Rees J, Cranston K (2017). Automated assembly of a reference taxonomy for phylogenetic data synthesis. Biodivers Data J.

[CR97] Reynolds PL, Bruno JF (2013). Multiple predator species alter prey behavior, population growth, and a trophic cascade in a model estuarine food web. Ecol Monogr.

[CR66] Roche DP, McGhee KE, Bell AM (2012). Maternal predator-exposure has lifelong consequences for offspring learning in threespined sticklebacks. Biol Lett.

[CR67] Rohatgi A (2020) WebPlotDigitizer, Pacifica, CA. https://automeris.io/WebPlotDigitizer

[CR68] Samia DSM, Nomura F, Blumstein DT (2013). Do animals generally flush early and avoid the rush? A meta-analysis. Biol Lett.

[CR69] Schmitz OJ, Barbosa P, Castellanos I (2005). Behavior of predators and prey and links with population-level processes. Ecology of predator-prey interactions.

[CR70] Schmitz OJ, Beckerman AP, O’Brien KM (1997). Behaviorally mediated trophic cascades: effects of predation risk on food web interactions. Ecology.

[CR71] Seckl JR (2004). Prenatal glucocorticoids and long-term programming. Eur J Endocrinol Eur Fed Endocr Soc.

[CR72] Sentis A, Hemptinne J-L, Brodeur J (2017). Non-additive effects of simulated heat waves and predators on prey phenotype and transgenerational phenotypic plasticity. Glob Change Biol.

[CR73] Sharda S, Zuest T, Erb M, Taborsky B (2021). Predator-induced maternal effects determine adaptive antipredator behaviors via egg composition. Proc Natl Acad Sci.

[CR74] Sheriff MJ, Love OP (2013). Determining the adaptive potential of maternal stress. Ecol Lett.

[CR75] Sheriff MJ, Thaler JS (2014). Ecophysiological effects of predation risk; an integration across disciplines. Oecologia.

[CR98] Sheriff MJ, Krebs CJ, Boonstra R (2009). The sensitive hare: sublethal effects of predator stress on reproduction in snowshoe hares. J Anim Ecol.

[CR76] Sheriff MJ, Krebs CJ, Boonstra R (2010). The ghosts of predators past: population cycles and the role of maternal programming under fluctuating predation risk. Ecology.

[CR77] Sheriff MJ, Dantzer B, Love OP, Orrock JL (2018). Error management theory and the adaptive significance of transgenerational maternal-stress effects on offspring phenotype. Ecol Evol.

[CR78] Sheriff MJ, Peacor SD, Hawlena D, Thaker M (2020). Non-consumptive predator effects on prey population size: a dearth of evidence. J Anim Ecol.

[CR79] Shine R, Downes SJ (1999). Can pregnant lizards adjust their offspring phenotypes to environmental conditions?. Oecologia.

[CR80] Snell-Rood EC, Swanson EM, Young RL (2015). Life history as a constraint on plasticity: developmental timing is correlated with phenotypic variation in birds. Heredity.

[CR81] Stamps JA, Krishnan VV (2017). Age-dependent changes in behavioural plasticity: insights from Bayesian models of development. Anim Behav.

[CR82] St-Cyr S, Abuaish S, Sivanathan S, McGowan PO (2017). Maternal programming of sex-specific responses to predator odor stress in adult rats. Horm Behav.

[CR83] Storm JJ, Lima SL (2010). Mothers forewarn offspring about predators: a transgenerational maternal effect on behavior. Am Nat.

[CR84] Tariel J, Plénet S, Luquet É (2020). Transgenerational plasticity in the context of predator-prey interactions. Front Ecol Evol.

[CR85] Uller T (2008). Developmental plasticity and the evolution of parental effects. Trends Ecol Evol.

[CR86] Uller T, Royle NJ, Smiseth PT, Kölliker M (2012). Parental effects in development and evolution. The evolution of parental care.

[CR87] Uller T, Nakagawa S, English S (2013). Weak evidence for anticipatory parental effects in plants and animals. J Evol Biol.

[CR88] Urban MC (2007). Risky prey behavior evolves in risky habitats. Proc Natl Acad Sci.

[CR89] Vassallo BG, Paitz RT, Fasanello VJ, Haussmann MF (2014). Glucocorticoid metabolism in the in ovo environment modulates exposure to maternal corticosterone in Japanese quail embryos (*Coturnix japonica*). Biol Lett.

[CR90] Viechtbauer W (2010). Conducting meta-analyses in R with the metafor Package. J Stat Softw.

[CR91] Walsh MR, Cooley F, Biles K, Munch SB (2015). Predator-induced phenotypic plasticity within- and across-generations: a challenge for theory?. Proc R Soc B Biol Sci.

[CR99] Ward AJ, Mehner T (2010). Multimodal mixed messages: the use of multiple cues allows greater accuracy in social recognition and predator detection decisions in the mosquitofish, Gambusia holbrooki. Behav Ecol.

[CR92] Wilson AJ, Réale D (2006). Ontogeny of additive and maternal genetic effects: lessons from domestic mammals. Am Nat.

[CR93] Wingfield JC, Maney DL, Breuner CW (1998). Ecological bases of hormone-behaviour interactions: the “emergency life history stage”. Am Zool.

[CR94] Wolf JB, Wade MJ (2016). Evolutionary genetics of maternal effects. Evolution.

[CR95] Yin J, Zhou M, Lin Z (2019). Transgenerational effects benefit offspring across diverse environments: a meta-analysis in plants and animals. Ecol Lett.

