# Supplementary material for: Predator-induced transgenerational plasticity in animals: a meta-analysis
Source: Oecologia. 2022 Nov 1;200(3-4):371–83. doi: 10.1007/s00442-022-05274-w (PMC9675678; doi:10.1007/s00442-022-05274-w)
Supplement: Supplementary file 1 — Supplementary file1 (DOCX 334 KB) [file 442_2022_5274_MOESM1_ESM.docx]

S1. PRISMA (Preferred Reporting Items for Systematic Reviews and Meta-Analyses) diagram showing the protocol and results of our systematic literature review.


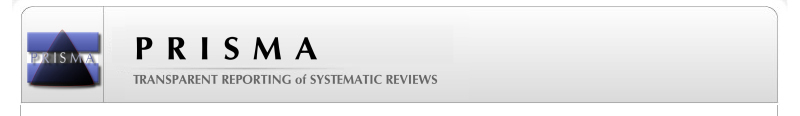


**P PRISMA flow diagram**

## Screening

## Included

## Eligibility

## Identification

Records identified through database searching
(n = 603 from WoS)

Additional records identified through other sources
(n = 42)

645 papers

Records after duplicates removed
(n = 636)

Records screened
(n = 636)

Records excluded
(n =465)

Full-text articles assessed for eligibility
(n = 171)

Full-text articles excluded, with reasons
(n = 122)

Studies included in qualitative synthesis
(n = 49)

Studies included in quantitative synthesis (meta-analysis)
(n = 49)

Selection criteria: We had four main criteria to select papers: 1) subjects were not domesticated species; 2) some aspect of next-generation offspring individual phenotype was measured (i.e. excluding grandparent-grand offspring effects i.e. if a study included both grandparental and parental effects, we only extracted parents-offspring effects); 3) parents and/or embryo were exposed to a predation risk and any effects were generated by predator exposure or cues, and not by direct manipulation of “stress”: i.e. treatment with glucocorticoids; and 4), subjects were exposed to predator cues in the context of breeding (i.e. not including studies of the effects of early- or mid-life predator exposure on reproduction) and with a controlled experiment (i.e. controlled and treatment groups, known predator for an observational study).

Exclusion of studies:

- **abstract screening level**: Two papers did not match the 1^st^ inclusion criterion as studies were carried out with domesticated species. 383 papers did not match the 2^nd^ criterion: 361 did not show any transgenerational effects from parents to offspring, 6 did not measure offspring traits, 16 did not provide individual phenotypic results (i.e. trait responses were measured only at the population- or clutch-level). 63 papers did not match the 3^rd^ criterion: 25 did not have any predation risk, 38 did not present any parental predator exposure. 5 papers did not match the 4^th^ criterion as they either did not have any control treatment, or the study was observational with non-identified predator(s), or offspring were exposed to predator in adulthood. Finally, it was impossible to get the required information in 12 papers (two were impossible to find on database searching and other sources, eight were reviews and two were conference abstracts).
- **full paper read level**: Five papers did not match the 1^st^ criterion as they were carried out on domesticated species (e.g. laboratory rodent or primate strains). The 2^nd^ criterion was not reached in 19 papers as the results were provided at the populational-level or only for parents without mentioning the results for offspring. 35 papers did not match our 3^rd^ criterion as they did not present any parental predator exposure. 18 papers did not match our 4^th^ criterion (predator exposure while offspring are independent, observational study with no identified predator and no control group, impossibility to disentangle mixed-up effects such as predation risk and cannibalism). Moreover, it was not possible to get the required information in 27 papers: 21 had missing data (i.e. impossibility to calculate the effect size because the sample size, SD, etc. was missing) and 6 papers were unavailable (i.e. full paper not available in English or the full paper was not available on the database searching (solely the abstract was).

S2. Trait categorisation information

Highlighted in yellow – traits for which the sign of the effect size was changed – these represent INCREASES in a trait that are biologically “negative” – i.e. so we can say whether overall parental pred exposure has a “positive” or “negative” effect.

| Traits | Binned category |
| --- | --- |
| \| \| distance to predator latency to first begin moving/enter chamber learning to find food preferred temperature dispersal probability/dispersal distance activity level proportion of time spent frozen tongue flicks locomotor speed time spent foraging/hiding etc anti-predator response left-turning preference space use metrics nearest-neighbour distance play attack frequency open field/maze test responses line-crossing total distance travelled number of rearing postures \| \| --- \| \| \| --- \| --- \| | performance/behaviour |
| \| carapace/rostra/shell length limb length sternum length wing length spine length total/partial body size measurements larval/birth/juvenile mass etc tail length:body length eye size mass/size at life history stage (e.g. first reproduction, hatching, fledging) body condition metrics muscle depth dry weight organ (e.g. testis) index tissue mass \| \| --- \| | size_mass |
| \| growth rate measure larval growth (length corrected for egg size) change in mass over expt age at fledging/maturation development time survival hatching time tissue growth \| \| --- \| | growth_development |
| \| baseline opercular beat rate baseline or stress-induced CORT/ACTH resting potential of mitral cells/mitral cell activity likelihood of being winged leukocyte count phtyohemagglutinin Challenge response respiration rate glucose \| \| --- \| | physiology |

S3.


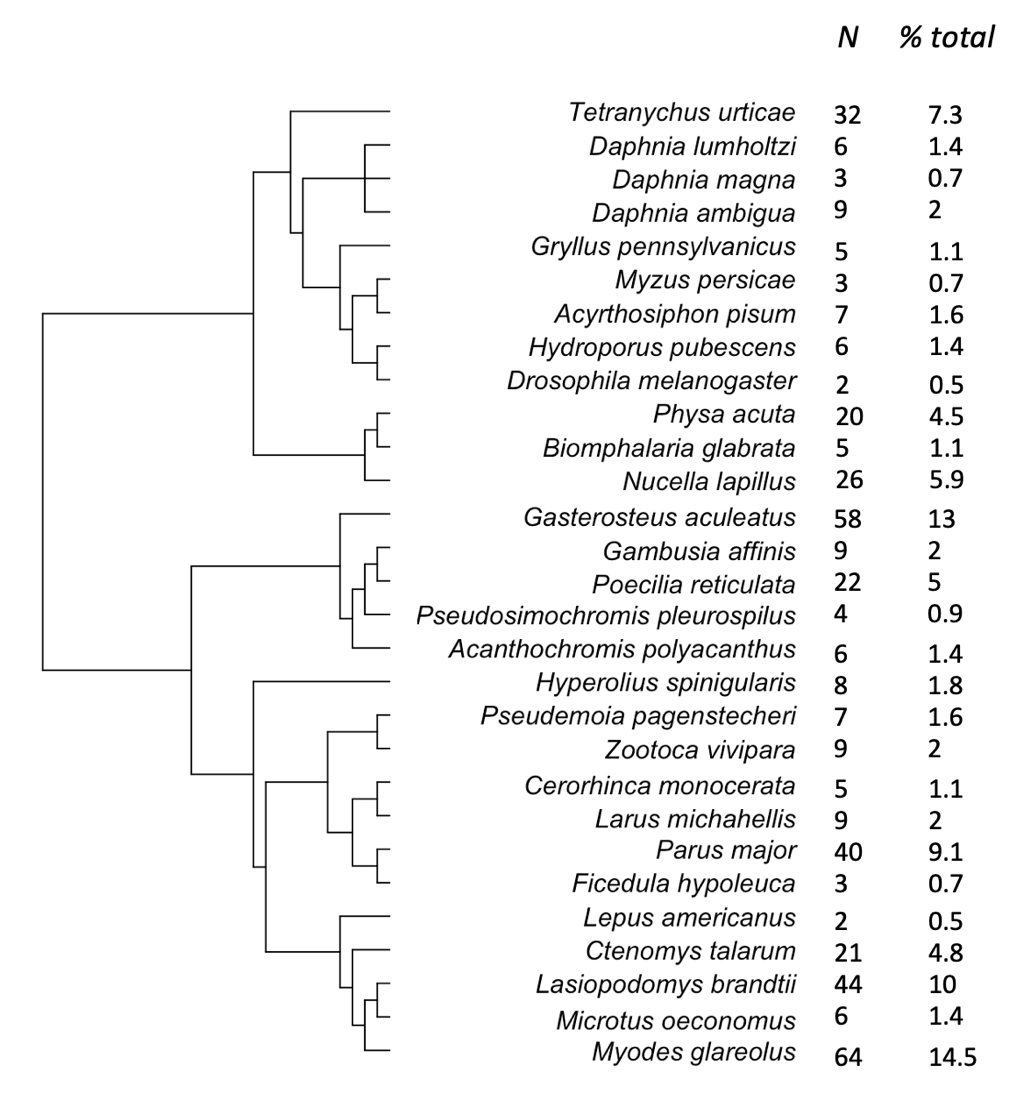


S3. Phylogenetic tree showing the representation of all the species included in meta-analytical models from the 49 studies we used. N represents the individual effect sizes associated with that species, and % total represents the percentage of the total effect sizes (N = 441).

S4. Sensitivity analyses

a) FULL MODEL: coefficients derived from robust variance estimation.

|  | **Est ± s.e.** | **T** | **P** | **95% CI (ci.lb, ci.ub)** |
| --- | --- | --- | --- | --- |
| *intercept* | *1.13 ± 0.42* | *2.70* | *0.01* | *0.24, 1.98* |
| Offspring trait |  |  |  |  |
| behaviour/performance |  |  |  |  |
| growth/development | 0.53 ± 0.63 | 0.85 | 0.40 | -0.74, 1.81 |
| physiology | -0.16 ± 0.47 | -0.35 | 0.73 | -1.12, 0.80 |
| size/mass | -0.46 ± 0.23 | -2.00 | 0.05 | -0.92, 0.01 |
| Age at measurement |  |  |  |  |
| embryo |  |  |  |  |
| birth | 0.06 ± 0.22 | 0.27 | 0.79 | -0.39, 0.52 |
| juvenile | -0.22 ± 0.18 | -1.21 | 0.24 | -0.59, 0.15 |
| maturity | 0.03 ± 0.22 | 0.12 | 0.90 | -0.41, 0.47 |
| Reproductive mode |  |  |  |  |
| oviparous |  |  |  |  |
| viviparous | 0.48 ± 0.60 | 0.81 | 0.42 | -0.73, 1.70 |
| Cue type |  |  |  |  |
| auditory |  |  |  |  |
| chemical | -1.36 ± 0.57 | -2.36 | 0.02 | -2.53, -0.19 |
| visual | -0.76 ± 0.30 | -2.53 | 0.02 | -1.36, -0.15 |
| multicomponent | -0.71 ± 0.31 | -2.32 | 0.02 | -1.33, -0.09 |
| predator presence | -0.79 ± 0.35 | -2.28 | 0.03 | -1.50, -0.09 |

b) OFFSPRING RISK ENVIRONMENT: influence on PiTGP, i) real coefficients, ii) coefficients derived from robust variance estimation.

|  | Est ± s.e. | T | P | **95% CI (ci.lb, ci.ub)** |
| --- | --- | --- | --- | --- |
|  |  |  |  |  |
| i) *intercept* | *0.12* | *-2.00* | *0.05* | *-0.23, -0.01* |
| Risk Environment  low risk |  |  |  |  |
| high risk | 0.10 | 1.34 | 0.18 | -0.05, 0.24 |
| ii) *intercept* | *-0.12* | *-2.64* | *0.02* | *-0.21, 0.02* |
| Risk Environment  low risk |  |  |  |  |
| high risk | 0.09 | 1.44 | 0.18 | -0.05 0.25 |

S5. Complete bibliography of all studies included in the meta-analysis

Mommer, B. C. & Bell, A. M. Maternal Experience with Predation Risk Influences Genome-Wide Embryonic Gene Expression in Threespined Sticklebacks (Gasterosteus aculeatus). PLOS ONE 9, e98564–e98564 (2014).

Basso, A., Coslovsky, M. & Richner, H. Parasite- and predator-induced maternal effects in the great tit (Parus major). Behavioral Ecology 25, 1105–1114 (2014).

Stratmann, A. & Taborsky, B. Antipredator defences of young are independently determined by genetic inheritance, maternal effects and own early experience in mouthbrooding cichlids. Functional Ecology 28, 944–953 (2014).

Roche, D. P., McGhee, K. E. & Bell, A. M. Maternal predator-exposure has lifelong consequences for offspring learning in threespined sticklebacks. Biol. Lett. 8, 932–935 (2012).

Sheriff, M. J., Krebs, C. J. & Boonstra, R. The sensitive hare: sublethal effects of predator stress on reproduction in snowshoe hares. The Journal of animal ecology 78, 1249–58 (2009).

Feng, S., McGhee, K. E. & Bell, A. M. Effect of maternal predator exposure on the ability of stickleback offspring to generalize a learned colour–reward association. Animal Behaviour 107, 61–69 (2015).

Bestion, E., Teyssier, A., Aubret, F., Clobert, J. & Cote, J. Maternal exposure to predator scents: Offspring phenotypic adjustment and dispersal. Proceedings of the Royal Society B: Biological Sciences 281, (2014).

Mommer, B. C. & Bell, A. M. A test of maternal programming of offspring stress response to predation risk in threespine sticklebacks. Physiology & Behavior 122, 222–227 (2013).

McGhee, K. E., Pintor, L. M., Suhr, E. L. & Bell, A. M. Maternal exposure to predation risk decreases offspring antipredator behaviour and survival in threespined stickleback. Functional Ecology 26, 932–940 (2012).

Coslovsky, M. & Richner, H. Predation risk affects offspring growth via maternal effects. Functional Ecology 25, 878–888 (2011).

Storm, J. J. & Lima, S. L. Mothers Forewarn Offspring about Predators: A Transgenerational Maternal Effect on Behavior. The American Naturalist 175, 382–390 (2010).

Dzialowski, A. R., Lennon, J. T., O’Brien, W. J. & Smith, V. H. Predator-induced phenotypic plasticity in the exotic cladoceran Daphnia lumholtzi. Freshwater Biology 48, 1593–1602 (2003).

Shine, R. & Downes, S. J. Can pregnant lizards adjust their offspring phenotypes to environmental conditions? Oecologia 119, 1–8 (1999).

Basso, A. & Richner, H. Predator-Specific Effects on Incubation Behaviour and Offspring Growth in Great Tits. PLoS ONE 10, e0121088 (2015).

Donelan, S. . C. & Trussell, G. C. Parental effects enhance risk tolerance and performance in offspring. Ecology 96, 2049–2055 (2015).

Harfenist, A. & Ydenberg, R. C. Parental provisioning and predation risk in rhinoceros auklets (Cerorhinca monocerata): effects on nestling growth and fledging. Behavioral Ecology 6, 82–86 (1995).

Freinschlag, J. & Schausberger, P. Predation risk-mediated maternal effects in the two-spotted spider mite, Tetranychus urticae. Exp Appl Acarol 69, 35–47 (2016).

Brodin, T., Johansson, F. & Bergsten, J. Predator related oviposition site selection of aquatic beetles (Hydroporus spp.) and effects on offspring life-history. Freshwater Biol 51, 1277–1285 (2006).

Plautz, S. C., Guest, T., Funkhouser, M. A. & Salice, C. J. Transgenerational cross-tolerance to stress: parental exposure to predators increases offspring contaminant tolerance. Ecotoxicology 22, 854–861 (2013).

Mikulski, A. & Pijanowska, J. When and how can Daphnia prepare their offspring for the threat of predation? Hydrobiologia 643, 21–26 (2010).

Basso, A. & Richner, H. Effects of nest predation risk on female incubation behavior and offspring growth in great tits. Behav Ecol Sociobiol 69, 977–989 (2015).

Giesing, E. R. Mothers transfer information via eggs: effect of mothers’ experience with predators on offspring. (University of Illinois at Urbana-Champaign, 2010).

Stein, L. R. & Bell, A. M. Paternal programming in sticklebacks. Animal behaviour 95, 165–171 (2014).

Vonesh, J. R. Egg predation and predator-induced hatching plasticity in the African reed frog, Hyperolius spinigularis. Oikos 110, 241–252 (2005).

Bian, J., Wu, Y. & Liu, J. Effect of predator-induced maternal stress during gestation on growth in root voles Microtus oeconomus. Acta Theriologica 50, 473–482 (2005).

Elliott, K. H., Betini, G. S., Dworkin, I. & Norris, D. R. Experimental evidence for within- and cross-seasonal effects of fear on survival and reproduction. Journal of Animal Ecology 85, (2016).

Morales, J., Lucas, A. & Velando, A. Maternal programming of offspring antipredator behavior in a seabird. Behavioral Ecology 29, 479–485 (2018).

Sentis, A., Hemptinne, J.-L. & Brodeur, J. Non-additive effects of simulated heat waves and predators on prey phenotype and transgenerational phenotypic plasticity. Glob Change Biol 23, 4598–4608 (2017).

Gu, C. et al. Effects of maternal stress induced by predator odors during gestation on behavioral and physiological responses of offspring in Brandt’s vole ( Lasiopodomys brandtii ). Integrative Zoology 13, 723–734 (2018).

Walsh, M. R. et al. Local adaptation in transgenerational responses to predators. Proc. R. Soc. B. 283, 20152271 (2016).

Sniegula, S., Nsanzimana, J. d’Amour & Johansson, F. Predation risk affects egg mortality and carry over effects in the larval stages in damselflies. Freshw Biol fwb.13261 (2019) doi:10.1111/fwb.13261.

Donelan, S. C. & Trussell, G. C. Synergistic effects of parental and embryonic exposure to predation risk on prey offspring size at emergence. Ecology 99, 68–78 (2018).

Donelan, S. C. & Trussell, G. C. Parental and embryonic experiences with predation risk affect prey offspring behaviour and performance. Proc. R. Soc. B. 285, 20180034 (2018).

Cattelan, S. et al. Maternal predation risk increases offspring’s exploration but does not affect schooling behavior. Behavioral Ecology 31, 1207–1217 (2020).

Atherton, J. A. & McCormick, M. I. Parents know best: transgenerational predator recognition through parental effects. PeerJ 8, e9340 (2020).

Sievert, T. et al. In utero behavioral imprinting to predation risk in pups of the bank vole. Behav Ecol Sociobiol 74, 13 (2020).

Ord, J., Holmes, K. E., Holt, W. V., Fazeli, A. & Watt, P. J. Premature birth stunts early growth and is a possible driver of stress‐induced maternal effects in the guppy POECILIA RETICULATA . J Fish Biol 96, 506–515 (2020).

Goeppner, S. R., Roberts, M. E., Beaty, L. E. & Luttbeg, B. Freshwater snail responses to fish predation integrate phenotypic plasticity and local adaptation. Aquat Ecol 54, 309–322 (2020).

McGhee, K. E., Paitz, R. T., Baker, J. A., Foster, S. A. & Bell, A. M. Effects of predation risk on egg steroid profiles across multiple populations of threespine stickleback. Sci Rep 10, 5239 (2020).

Hu, L., Gui, W., Chen, B. & Chen, L. Transcriptome profiling of maternal stress‐induced wing dimorphism in pea aphids. Ecol Evol 9, 11848–11862 (2019).

Li, G.-Y. & Zhang, Z.-Q. Development, lifespan and reproduction of spider mites exposed to predator-induced stress across generations. Biogerontology 20, 871–882 (2019).

Sentis, A. et al. Different phenotypic plastic responses to predators observed among aphid lineages specialized on different host plants. Sci Rep 9, 9017 (2019).

Gu, C. et al. Effects of predator-induced stress during pregnancy on reproductive output and offspring quality in Brandt’s voles (Lasiopodomys brandtii). Eur J Wildl Res 66, 14 (2020).

Lehto, W. R. & Tinghitella, R. M. Predator‐induced maternal and paternal effects independently alter sexual selection. Evolution 74, 404–418 (2020).

Monteforte, S., Cattelan, S., Morosinotto, C., Pilastro, A. & Grapputo, A. Maternal predator‐exposure affects offspring size at birth but not telomere length in a live‐bearing fish. Ecol Evol 10, 2030–2039 (2020).

Tariel, J., Plénet, S. & Luquet, É. Transgenerational plasticity of inducible defences: Combined effects of grand‐parental, parental and current environments. Ecol Evol 10, 2367–2376 (2020).

Hahn, M. A. & Von Elert, E. The impact of diel vertical migration on fatty acid patterns and allocation in Daphnia magna. PeerJ 8, e8809 (2020).

Lehto, W. R. & Tinghitella, R. M. Joint maternal and paternal stress increases the cortisol in their daughters’ eggs. Evolutionary Ecology Research 20, 133–144 (2019).

Morosinotto, C., Thomson, R. L., Korpimäki, E., Mateo, R. & Ruuskanen, S. Maternal food supplementation and perceived predation risk modify egg composition and eggshell traits but not offspring condition. J Exp Biol 222, jeb201954 (2019).
